# Study on Grouting Performance Optimization of Polymer Composite Materials Applied to Water Plugging and Reinforcement in Mines

**DOI:** 10.3390/ma17174245

**Published:** 2024-08-28

**Authors:** Xuanning Zhang, Ende Wang, Sishun Ma, Deqing Zhang

**Affiliations:** Department of Geology, College of Resources and Civil Engineering, Northeastern University, Shenyang 110819, Chinamasishun@stumail.neu.edu.cn (S.M.); 1910353@stu.neu.edu.cn (D.Z.)

**Keywords:** grouting, polyurethane, composites, mechanical properties, modification

## Abstract

With the increasing drilling depth of mines, the cross-complexity of fissures in the rock body, and the frequent occurrence of sudden water surges, polymer slurry, with its advantages of good permeability and strong water plugging, is increasingly used in mine grouting projects. Additional research is needed in order to further improve the grouting performance of polymer slurry, ensure the safety of mining operations, and reduce the grouting cost. In this paper, a polymer composite grouting material was prepared with diphenyl methyl diisocyanate, polyether polyol, and fly ash, as the main raw materials, with coupling agent and catalyst as auxiliary reagents. The performance of the composite grouting material in terms of mechanical properties, thermal stability, hydrophobicity, and bonding was explored. This study’s findings indicated that incorporating fly ash led to notable enhancements in the thermal stability and water resistance of the polymer slurry. Furthermore, the introduction of fly ash notably raised the starting degradation temperature of the polymer, boosted the water contact angle of the composite material, and reduced the density and reaction temperature of the composite material. In addition, the catalyst and coupling agent as auxiliary reagents affected the polymers in terms of mechanical properties; in this paper, dibutyltin dilaurate was used as the catalyst, and organosilanes were used as the coupling agent. The catalyst successfully sped up the polymer’s gel time, however, an excessive quantity of catalyst compromised the polymer’s mechanical characteristics. The addition of organosilanes has a positive effect on the dynamic mechanical properties of the composites, fracture toughness, compression, bending, and bond strength. The research can offer a theoretical direction for creating polymer mixtures in mine grouting projects.

## 1. Introduction

The nation’s economic growth and social advancement are greatly reliant on its mineral resources, and the important way to more effectively encourage the rapid expansion of the national economy is to consistently improve the development and usage efficiency of these resources [1,2,3]. However, as a result of extensive natural resource exploitation, surface resources are no longer sufficient to meet societal demands, which is driving up the intensity of underground mining [4,5]. The complex and varied geological circumstances in deep mine mining can lead to a tendency for the rock body to become loose during the operation [6,7,8]. This loose rock body increases the likelihood of mishaps, such as unexpected water leakage, fissure rockslides, and working face collapse [9,10]. Construction operations have a certain amount of risk, and if a safety incident happens, it could jeopardize public safety while also causing significant losses to national property. The regular operation of a mining project depends on the application of a range of cutting-edge technical techniques to prevent and control potential dangers in the mine production process [11,12,13,14]. This will increase mining efficiency and safety. Water conservation, transportation, mining, and other engineering domains have all made extensive use of grouting technology, a significant water plugging and reinforcement technique [1,15,16]. Studies on grouting materials used in mining engineering are becoming more and more numerous. Following decades of research and development, scientists have created an increasing number of lightweight organic polymer grouting materials. Among these, polyurethane grouting material is thought to be environmentally friendly and is being used more and more in the mining industry and other related fields [17,18]. For example, polyurethane ballast materials can improve the bearing capacity of the ballast bed, reduce the subsidence deformation of the ballast bed, improve the longitudinal and horizontal resistance of the ballast bed, and enhance the stability of the ballast bed. The polyurethane solidified track bed technology originated from Bayer and has been laid on China’s high-speed railway lines since 2009. In 2018, the 6-km polyurethane solidified track bed experimental line laid on Jiqing High-speed Railway was successfully opened to traffic, at a speed of 350 km per hour.

Currently, inorganic and organic chemical grouting materials are available in significant numbers for usage. Conventional grouting reinforcement materials, such as cement, water glass, and clay, have clear disadvantages as compared to organic chemical grouting materials, despite their low cost, non-toxicity, and high solid body strength [19,20,21]. For instance, the huge particle size of inorganic materials, like cement, limits their capacity to penetrate and makes it challenging to get into small-pore cracks. The range of applications for inorganic grouting materials, like cement, is also severely restricted by the relatively poor bonding qualities, ease of erosion by water flow, and difficulty in precisely regulating or controlling the gel formation time. Researchers are becoming more and more interested in chemical grouting materials since they are extremely effective reinforcing and waterproofing materials [5,8,22]. A multitude of novel polymer grouting materials are being developed as science and technology advance. Common chemical grouting material kinds include lignin, epoxy resin, polyurethane, and acrylamide series. Among these, polyurethane grouting material has gained a lot of attention in recent years due to its excellent durability and water resistance. One type of polymer chemical grouting material that can effectively stop water leaks and reinforce structures quickly is polyurethane [23,24]. Among all organic grouting materials, polyurethane-based grouting materials are used in engineering applications the most frequently because of their exceptional all-around performance.

Grouting materials based on polymers, such as polyurethane, have become increasingly popular due to the steady rise in demand for organic polymers. In recent years, numerous academics have conducted a great deal of research in this field. A water-glass polyurethane composite material was made by Hong et al. [25]. The composite material exhibited good mechanical properties and considerably enhanced tensile, bond, and compressive strengths when the water-glass mass fraction reached 25%. Lu et al. [26] developed a bio-based polymer grouting material and assessed its mechanical, anti-slip, and water permeability qualities using regenerated ceramic aggregates rather than natural aggregates. According to S. Samaila et al. [9], the grouting effect of polyurethane slurry was significantly influenced by the size of the pores in the cracks and the gravel’s particle size. The polyurethane slurry penetrates the weak soil by filling in existing cracks and creating new ones by following the path of least resistance. As it spreads into the larger soil fissures, the mechanical properties of the weak soil improve. Shi et al. [27]‘s investigation into the relationship between temperature and the compressive strength of polymer grouting material revealed that, for polyurethane materials with densities less than 0.4 g/cm^3^, temperature has a relatively small effect on the material’s compressive strength; however, for materials with densities greater than 0.4 g/cm^3^, the degree to which temperature affects the material’s mechanical properties should not be disregarded, and the material’s compressive strength decreases as temperature rises. When cyclic loading is applied to polyurethane material, Gao et al. [28] discovered that the material exhibits three distinct response phases. The study’s findings also indicate that polyurethane materials have a stress threshold for fatigue failure that lowers as polyurethane density increases. Monika et al. [29] reinforced the prepared rigid polyurethane foam by adding 5% and 10% fly ash to two different types of boilers, respectively. The foaming process, physical properties, morphology, and thermal degradation properties were compared and analyzed. Studies have shown that fly ash enhances the foam synthesis reaction, most commonly polyurethane foam with 10% PFA added. In addition, the addition of fillers affects the morphology of the material and reduces the brittleness of the material. Partial substitution of petrochemical components with waste fillers also reduces the total energy consumption in the production of polyurethane composites. Srihith et al. [30] used low-cost and environmentally friendly fly ash particles and silica particles as additives for rigid polyurethane foam composites. Through a series of experiments and tests on composite materials, the research results show that fly ash can significantly improve the mechanical and thermal properties of composite materials. When the weight percentage of additives is 10%, compared with the composite material loaded with silica, the total calorific value of the composite material loaded with fly ash is reduced by 12%, and the compressive strength is increased by 21%. The mechanical characteristics and penetration grouting capabilities of polyurethane gel materials have become better understood, thanks in large part to these investigations. Nevertheless, the expensive price of polymer materials prevents its widespread application. Fly ash is a cheap and chemically stable industrial byproduct. In addition to lowering the cost of engineering grouting, combining fly ash and polyurethane to create a composite grouting material and regulating the material’s grouting performance with catalysts and coupling agents, also enhances the physical characteristics of polymer slurry. There aren’t many studies being done in this field right now.

In this paper, taking the polyurethane-fly ash composite material as the main body, the effects of different dosages of fly ash and auxiliary reagents on the performance of the composite material were investigated through the test methods of compression test, bending test, adhesion test, dynamic mechanical test, thermogravimetric test, and contact angle test. The ideal dosage of each raw material of the composite material is proposed, which provides a new idea for the design of composite water plugging grouting material in mines.

## 2. Methodology

### 2.1. Materials

Isocyanate and either polyether polyol or polyester polyol are the primary components of polyurethane grouting materials, with fly ash serving as an additional raw material for modified polyurethane slurry. Catalysts and coupling agents are additional reagents that are crucial in influencing the physical characteristics of polyurethane. The main components and the proportion of fly ash are shown in Table 1.

The isocyanate and polyether polyol utilized in this study are sourced from Zhengzhou Sichuang Polyurethane Materials Co. Isocyanate, and appears black with a viscosity of 0.15 Pa·s and a mass fraction of 30% -NCO, while polyether polyol is brownish yellow with an -OH content of 55 mg KOH/g. Fly ash comes from Anda Environmental Protection Science and Technology Co., Ltd. located in Dongjiang Street, Yantai City, Shandong Province. The organotin catalyst was obtained from Runyou Chemical Co., Ltd. located in Futian Street, Futian District, Shenzhen, China. Organosilanes were obtained from Daoning Chemical Co., Ltd. located in Xuanwu District, Nanjing, China. The materials used, are shown in Table 2.

### 2.2. Preparation of Samples

**Figure 1:**
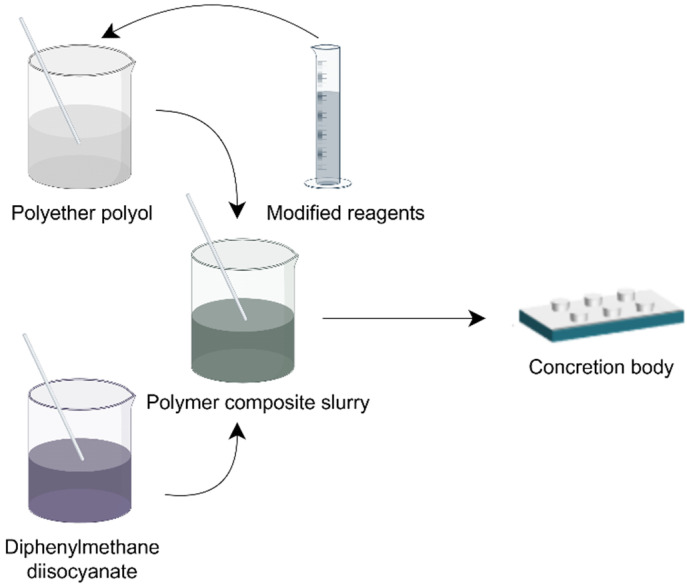
Schematic diagram of polymer composite slurry preparation.

Diphenylmethane diisocyanate (MDI) was placed in a beaker container labeled as solvent A. An amount of polyether polyol, catalyst, organosilane, and fly ash was placed in another beaker container labeled as solvent B. The solvents A and B were mixed and stirred homogeneously at a speed of 200 rpm for 5 min and then kept in a dry and sealed container for more than 20 min. A blend of polymers was poured into a PVC pipe mold measuring 50 × 100 mm, and treated with silicone oil release agent on the inner wall, to create a cylindrical sample for testing compression strength. The preparation process of the sample is shown in Figure 1. Additionally, the slurry was poured into a rectangular mold with dimensions of 50 mm length, 15 mm width, and 5 mm thickness to measure the dynamic mechanical properties of the sample. The whole process was kept at room temperature of 25 °C. The polymer gel material was left to cure for 1 h, and then the samples were removed from the molds and conditioned at room temperature and pressure to form standard specimens to be measured.

### 2.3. Characterization Techniques

The thermal stability of the polymer composite gel material was tested using a Mettler-Toledo TGA3 thermogravimetric analyzer from Zurich, Switzerland, in a nitrogen atmosphere. The temperature was programmed to increase from 35 to 700 °C, at a rate of 10 °C per minute.

In order to study the hydrophobicity of the polymer gel materials, a standard contact angle meter model DSA250 manufactured from KRUSS Company, Hamburg, Germany, was used to determine the contact angle of the samples. The surface of the sample needed to be wiped clean before measurement and the test was repeated 10 times to complete a set of data.

### 2.4. Mechanics Performance Testing

A thermodynamic dynamic analyzer, manufactured by TA-Waters, New Castle, DE, USA, was used to explore the dynamic mechanical properties of the polymer gel material in the temperature range of 20–200 °C. The test was conducted at a frequency of 1 Hz, with a temperature ramp rate of 10 °C per minute.

As per the method for Plastics-Determination of Compressive Properties [31], the samples were compressed with the WDW-20 universal testing machine manufactured by Kece Testing Technology Co. Ltd., located in Tianqiao District, Jinan, China. The compression rate was set to 20 mm/min, and the test was repeated five times for each set of data. The compressive strength expression is shown in Equation (1), where σ is the compressive strength in MPa, F is the instrumental pressure in N, and A is the original cross-sectional area of the specimen in mm^2^.

(1) $$\sigma =\frac{F}{A}$$

The samples were subjected to a three-point bending test using a universal testing machine to determine their flexure strength. The span was set to 70 mm and the loading rate of the testing machine was set to 2 mm/min. Equation (2) displays the formula for flexure strength, with σ_f_ representing the bending stress in MPa, F representing the applied force in N, L representing the span in mm, b representing the width of the sample in mm, and h representing the thickness of the sample in mm.

(2) $$\sigma_{f}=\frac{3FL}{2bh^{2}}$$

A universal testing machine was used to test the bond strength of the samples, according to the method of “Adhesives—Determination of Tensile Strength” [32]. The specimens were clamped symmetrically on a fixture, and the distance between the clamping place and the nearest bonding end was 50 mm. The testing machine was loaded at a constant rate, and the loading rate was set at 9 MPa/min.

## 3. Results and Discussion

### 3.1. Thermogravimetric Experiment

**Figure 2:**
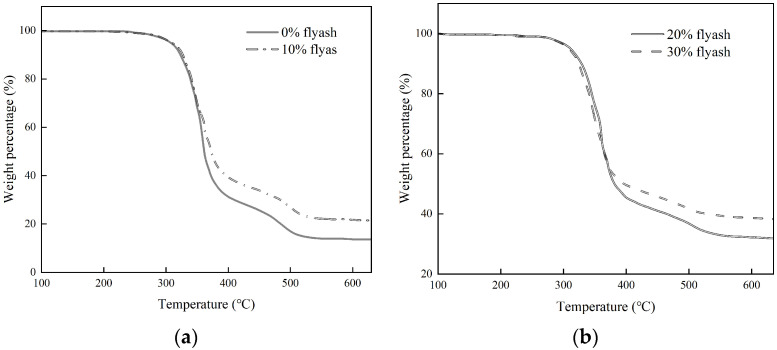
TG curves of different dosages. (**a**) The dosage of fly ash is 0 and 10%; (**b**) The dosage of fly ash is 20% and 30%.

The thermal stability of composite gel materials with different amounts of fly ash under a nitrogen atmosphere was studied by the thermogravimetric analyzer. The corresponding curves are shown in Figure 2.

Figure 2 illustrates the polymer gel material’s thermal degradation process, which consists of three distinct stages. The first stage occurs when the temperature is less than 270 °C. In this stage, the weight loss of the polymer gel material is small, which is mainly caused by the evaporation of water and other low-boiling-point substances, as well as the overflow of carbon dioxide. The second stage occurs when the temperature is between 280 °C and 390 °C. The weight loss in this stage is the largest, reaching 60% to 70%. The main reason for this is that at this stage, the hard chain segments of the polyurethane material molecular chain begin to degrade, including the decomposition of polyols and isocyanate components, which generate organic amines, hydrogen, methane, etc. The third stage occurs between 390 °C and 620 °C, in which the soft chain segments of the polyurethane molecular chain begin to degrade, and the overall degradation process is relatively slow.

By comparing varying levels of fly ash, it becomes evident that the temperature for polymer material degradation initiation rises with increased fly ash content, indicating that the inclusion of fly ash delays polymer material degradation and enhances its heat resistance. The enhanced heat resistance of the composite gel material may be attributed to the incorporation of fly ash, leading to interactions with the polyurethane gel’s soft chain segments. This interaction creates a barrier effect that hinders the escape of volatiles produced during polymer degradation. The presence of silica and metal oxides in fly ash helps create a dense carbon layer, which slows down the release of volatile degradation products and enhances the thermal stability of the polymer gel at this specific temperature.

The results of thermal stability tests of the polymer materials showed that the thermal degradation rate of the fly ash polymer composite gel material decreased with the increase of the fly ash dosage and the thermal stability was subsequently enhanced.

### 3.2. Hydrophobicity Analysis

**Figure 3:**
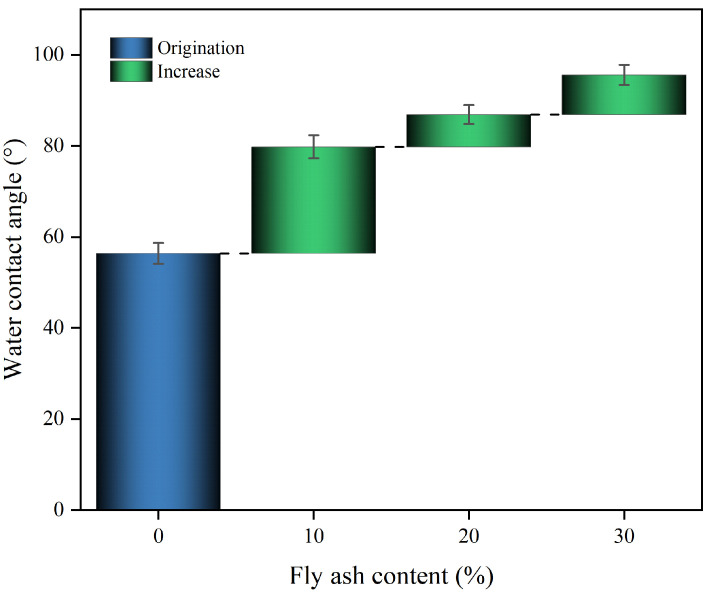
The variation of contact angle of composite materials with the content of fly ash.

The contact angle of polymer composites changes as fly ash content increases, as shown in Figure 3.

The contact angle of the composites gradually increases with the increase of fly ash dosage, but the growth decreases gradually. When the amount of fly ash is 30%, the contact angle of the composite material reaches 95.6°, which is 70% more than that of the pure polyurethane material. It can be seen that fly ash can significantly improve the hydrophobicity of the polymer gel material.

The reasons for this phenomenon may be, firstly, that the introduction of fly ash significantly increases the surface roughness of the polyurethane material. When the roughness is increased, capillary action enhances the droplet holding capacity, which results in insufficient droplet stretching on uneven surfaces, which often manifests itself as an increase in the contact angle. On the other hand, fly ash contains components such as silica and alumina, and the addition of these components changes the chemical properties of the polymer composite. Due to the low surface energy of silica and alumina, this reduces the hydrophilicity of the material surface, which in turn increases the contact angle.

### 3.3. Density and Reaction Temperature

**Figure 4:**
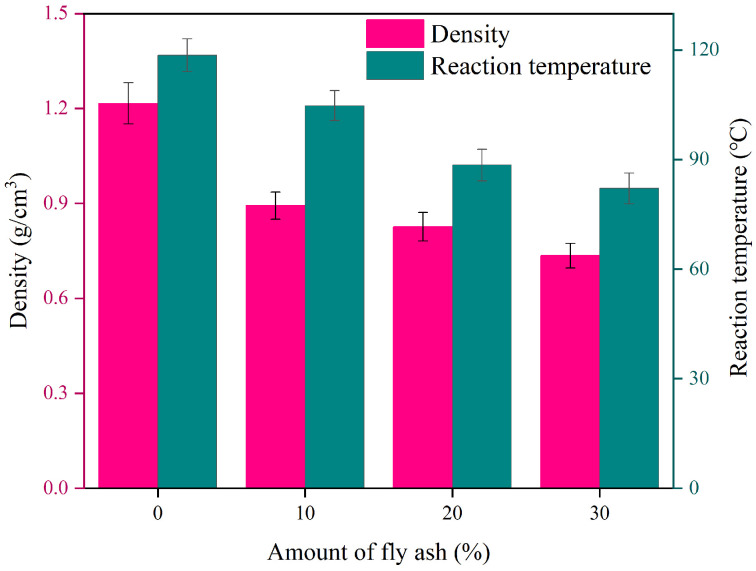
The variation of density and reaction temperature with the amount of fly ash.

In the process of filling grouting in mining engineering, the control of density and the reaction temperature in the process of slurry gel is very important in the whole grouting process. The low-density gel can better fill the cracks and pores, and the polymer gel curing process is an exothermic reaction, and too high reaction temperature poses a great threat to the safe production of underground mines. Figure 4 demonstrates the changing law of the density and reaction temperature of polymer composites with the amount of fly ash.

It is easy to see that the density and reaction temperature of the material decrease with the increase of fly ash. When the amount of fly ash is 30%, the density of the material decreases to 0.735 g/cm^3^, and the reaction temperature of the material decreases from 118 °C to 82 °C. The reason is that fly ash has a high thermal conductivity, as its micron and nanometer particles can significantly improve the overall thermal conductivity of the composite material so that the heat can be quickly transferred out through the matrix, and then realize the reduction of the reaction temperature. Furthermore, incorporating fly ash can enhance the uniformity of the polymer and decrease the occurrence of localized heat buildup, ultimately preventing overheating in specific areas and lowering the overall reaction temperature.

### 3.4. Dynamic Mechanical Analysis

**Figure 5:**
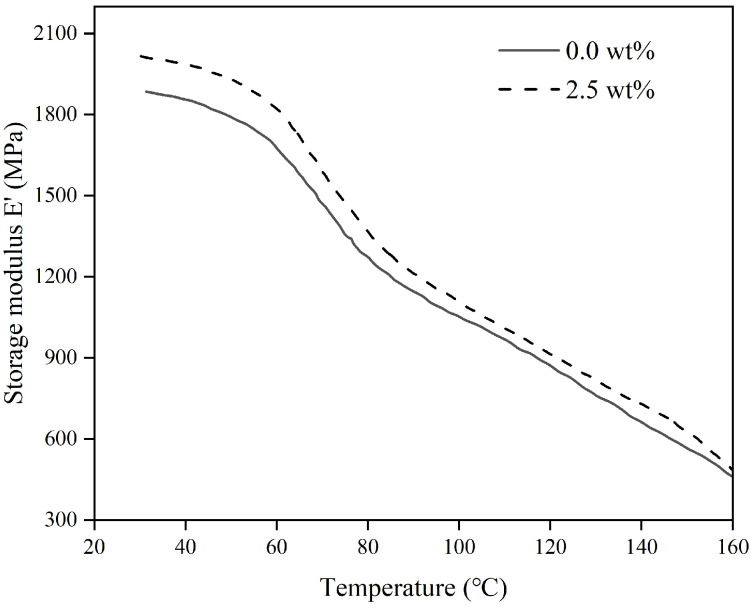
The variation of storage modulus with temperature under different dosages of coupling agents.

**Figure 6:**
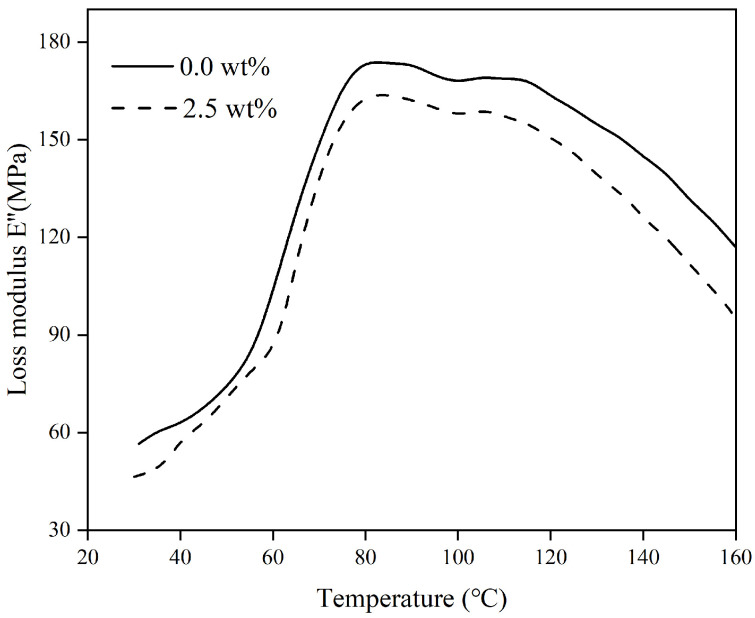
The variation of loss modulus with temperature under different dosages of coupling agents.

**Figure 7:**
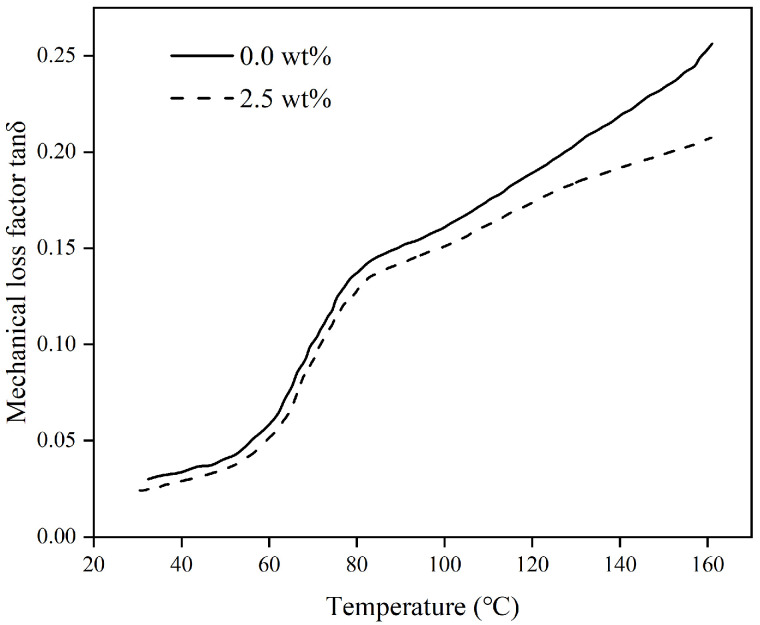
The variation of loss factor with temperature under different dosages of coupling agents.

Figure 5, Figure 6 and Figure 7 show how the energy storage modulus (E′), loss modulus (E″), and loss factor (tanδ) change with temperature in polymer composites containing varying levels of organosilanes.

The elastic modulus, or energy storage modulus, represents the capacity of a material to store energy through reversible elastic deformation during deformation, indicating the material’s resistance to deformation and stiffness. The greater the energy storage modulus, the greater the stiffness of the material. Figure 5 shows that the energy storage modulus of polymer composites with different organosilane contents has basically the same trend as the curve of temperature change, and when the temperature rises from 50 °C, the energy storage modulus decreases rapidly, indicating that the material begins to change from an amorphous state to glassy state. Conversely, incorporating organosilanes enhances the storage modulus of the composite gel material. The reason could be that the organosilane molecules can accelerate the cross-linking reaction between the polyurethane and the fly ash and promote the organic reaction, which improves the interfacial properties and cross-linking density of the material. As a result, the storage modulus values of specimens containing organosilanes rise more significantly than those without organosilanes when the materials are fully cured internally.

Loss modulus, also known as viscous modulus, refers to the size of energy lost due to irreversible viscous deformation of the material when deformation occurs, reflecting the viscous size and impact resistance of the material. Figure 6 demonstrates the variation rule of loss modulus (E″) of polymer composites with temperature.

Figure 6 illustrates that as the temperature rises, the loss modulus initially rises before quickly decreasing, peaking at around 80 °C. This indicates a shift in the movement of certain molecular chains in the non-crystalline area of the polymer material, transitioning from a rigid lattice-like structure to a more flexible state.

The loss factor, also referred to as the loss angular tangent (tanδ), is the ratio of the loss modulus to the energy storage modulus, indicating the viscoelastic properties of the material. As the loss factor increases, so does the energy loss and the internal energy generated. The glass transition temperature is the temperature at which a material changes from a highly elastic state to a glassy state, and the relaxation properties of a polymer material can be seen from the glass transition temperature. Polyurethane materials, which consist of hard and soft segment molecular chains in block copolymers, have a direct impact on the mechanical properties of polymer composites through the glass transition temperature of the hard segment molecular chains. Figure 7 shows the variation rule of the loss factor of the polymer composites with time.

Through Figure 7, it can be found that the loss factor gradually increases with the increase in temperature. This indicates that the higher the temperature, the greater the internal friction generated within the material, and the greater the energy loss. The loss factor curve reaches its highest point at temperatures ranging from 60 °C to 80 °C, indicating that this temperature range represents the glass transition temperature of the rigid portion of the polymer composite. When the composite material undergoes a glass transition, the kinetic energy of molecular motion increases during this period. After the barrier is broken, the moving units inside the molecules are activated and the movement of the molecular chain segments starts to occur. Furthermore, the polymer composites exhibit a higher glass transition temperature with the addition of organosilanes, as illustrated in Figure 7. The main reason for such a pattern is that the addition of organosilanes increases the crosslink density of the polymer composite and reduces the free volume of the composite, resulting in greater constraints on the molecular chains of the material. In addition, the average length of the molecular chains in neighboring crosslinking positions decreases, further hindering the movement of the molecular segments. On the other hand, it can also be seen from Figure 7 that the incorporation of organosilanes resulted in a longer range of glass transition temperatures for the polymer composites. The restriction of the molecular chains of the polyurethane by the organosilanes may explain the widening of the glass transition temperature of the polymer. Therefore, it can be concluded that organosilanes not only increase the energy storage modulus of the polymer composites, which in turn improves the stiffness of the materials but also have a positive effect on the improvement of the crosslink density of the materials.

### 3.5. Flexure Strength

**Figure 8:**
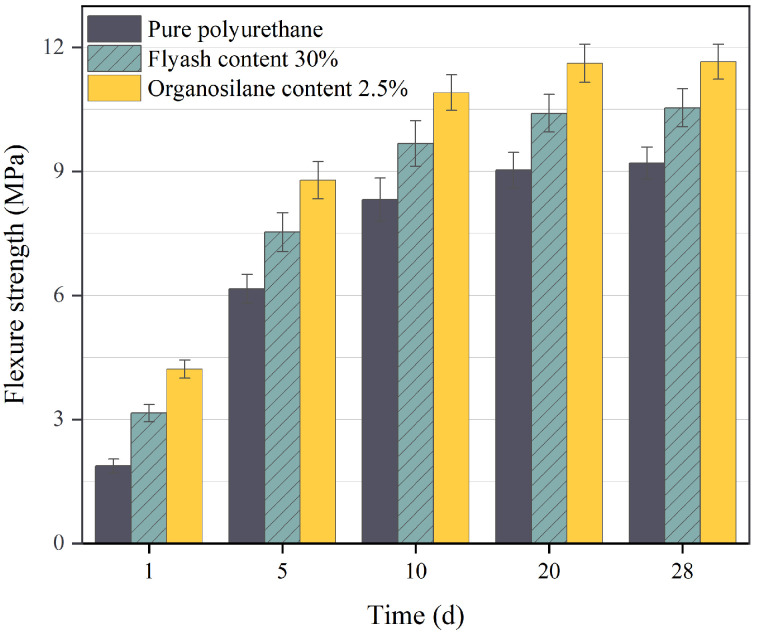
The variation of flexure strength with curing time under different polymer additives.

Figure 8 demonstrates the variation rule of flexural strength with curing time for pure polyurethane material, fly ash polyurethane composites, and composites with the addition of organosilanes. In Figure 8, it can be observed that there is a significant increase in the flexural strength of the material during the initial 5 days, followed by a slower growth rate between days 10 to 20.

Subsequently, the flexural strength stabilizes from day 20 to 28, indicating that the material has fully cured. The flexural strength of the fly ash polymer composites increased significantly as compared to the pure polyurethane material. The early flexural strength of the organosilane-added composites reached 4.2 MPa within 1 day of curing, which was 120% higher than that of the unmodified polyurethane material, and the flexural strength of the modified composites reached a maximum of 11.6 MPa after 28 days of curing, which was 27% higher than that of the pure polyurethane material. This indicates that the addition of organosilanes as well as fly ash to the polymer, can significantly improve the early strength as well as the final strength of the material. Adding fly ash increases material homogeneity and enhances compatibility between hard and soft polyurethane segments, leading to a more stable and uniform polymer phase structure. In addition, the organosilanes are dispersed in an orderly manner on the surface of the fly ash and within the organic phase, avoiding stress concentrations.

### 3.6. Fracture Toughness

**Figure 9:**
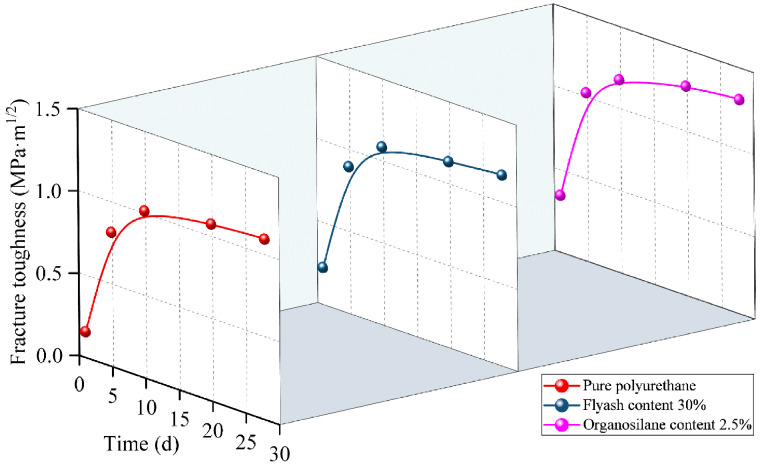
The variation of fracture toughness with curing time under different polymer additives.

The fracture toughness fluctuation patterns over time for pure polyurethane materials, fly ash-containing polyurethane composites, and organosilane-doped composites are displayed in Figure 9.

This can be seen in Figure 9, which illustrates how the fracture toughness of the three samples increases with curing time. For all three samples, the rate of increase in fracture toughness during the first five days is comparatively considerable. The fracture toughness of the organosilane-doped composite and the polymer composite with fly ash increased by 10% and 25.6%, respectively. After ten days, the samples’ fracture toughness essentially stopped increasing, and the fracture toughness of the organosilane-doped composite increased by 19.1%. The ability of polymer materials to absorb impact energy and their resistance to crack extension are the primary factors that determine how tough they are when subjected to impact loading. Fly ash can effectively mask the crack tip and establish a ductile crack bridge to the ductile particles by producing a plastic deformation zone at the crack tip when combined with the polymer matrix. The ductile particles and the polymer matrix work together to withstand the compressive stress on both sides throughout the stressing process, which stops the fracture from spreading further and increases the material’s toughness. Furthermore, the organosilanes create a silica oxide-rich interfacial layer on the fly ash surface. This layer can act as an extra chemical link between the fly ash and the polyurethane matrix, creating a strong interfacial bond that prevents cracks from propagating as quickly and increases the material’s fracture toughness.

### 3.7. Bonding Strength

**Figure 10:**
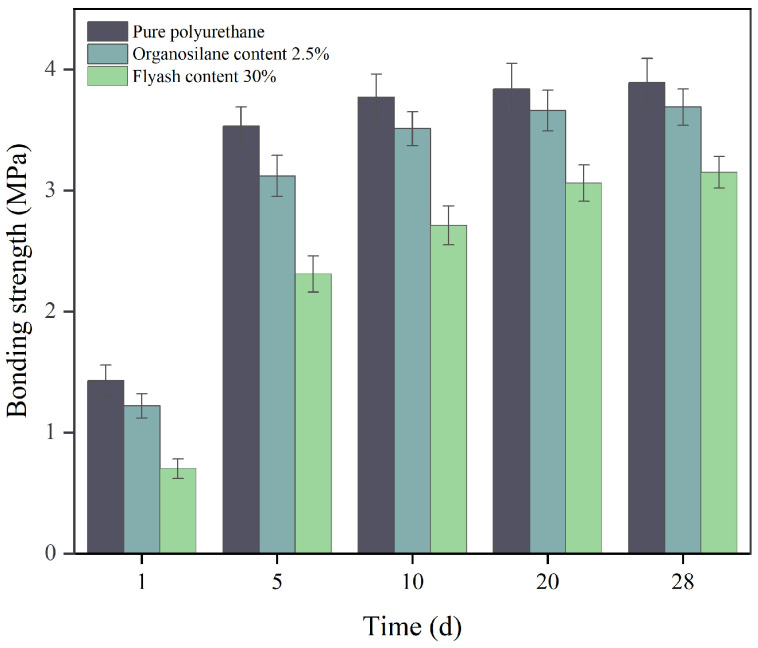
The variation of bonding strength with curing time under different polymer additives.

In Figure 10, the changes in bond strength over time are displayed for pure polyurethane materials, polyurethane composites with fly ash, and composites incorporating organosilanes.

Figure 10 shows that after 5 days of curing time, the polymer material fully undergoes a cross-linking reaction, which leads to a substantial increase in the bond strength of the material, but the growth rate of the bond strength of the material gradually slows down after that. Furthermore, following the incorporation of fly ash into the polyurethane substance, the adhesive power of the polymer notably declined, resulting in a maximum bond strength reduction of 3.15 MPa, representing a 32% decrease. The reason for such a result is that a series of chemical reactions accompany the polymer material during the solidification process from liquid to solid condensation. At the initial stage of the polymer reaction, the slurry forms a thermoplastic and soluble polymer with high bonding properties. Along with the continuation of the chemical reaction, the gelation process of the polymer ends when the molecular chain between the cross-linking points within the polymer reaches a certain length. At this time, the surface bonding strength of the polymer is reduced to zero, and the bonding strength is mainly maintained by the hard segments of the molecular chains inside the polyurethane. Incorporating fly ash causes the inorganic particles to function as rigid sections of the polyurethane molecular chain to some degree. Although it increases the stiffness of the material, the fly ash particles cannot cross-link with the molecular chain of the polyurethane, and it is difficult to form a stable chemical bond, so it cannot help the bond strength of the polymer material. The formation of the bond strength of the polymer can only rely on the pre-thermoplastic phase of the gelation process. For this reason, when fly ash is added to the polymer slurry, the fly ash restricts the formation of the bond strength of the polymer, resulting in a reduction in the bond strength of the composite material.

Conversely, Figure 10 illustrates that the inclusion of organosilanes in the composites led to a notable enhancement in bond strength, reaching a peak of 3.69 MPa, representing a 17% increase compared to composites lacking silanes. This is mainly because the addition of organosilanes generates new chemical bonds. The terminal molecular chains comprising the organosilanes reacted with the hydroxyl groups of the alcohols in the composites to produce hydrogen bonding, which was immediately followed by the dehydration and curing process of the material, which further produced covalent bonding. Simultaneously, the epoxy functionalities of the organosilanes form a strong connection with the polyurethane, enhancing the bond strength of the composites by combining these two strengths.

### 3.8. Gel Time and Compression Strength

#### 3.8.1. Effect of Catalyst on Composite Materials

**Figure 11:**
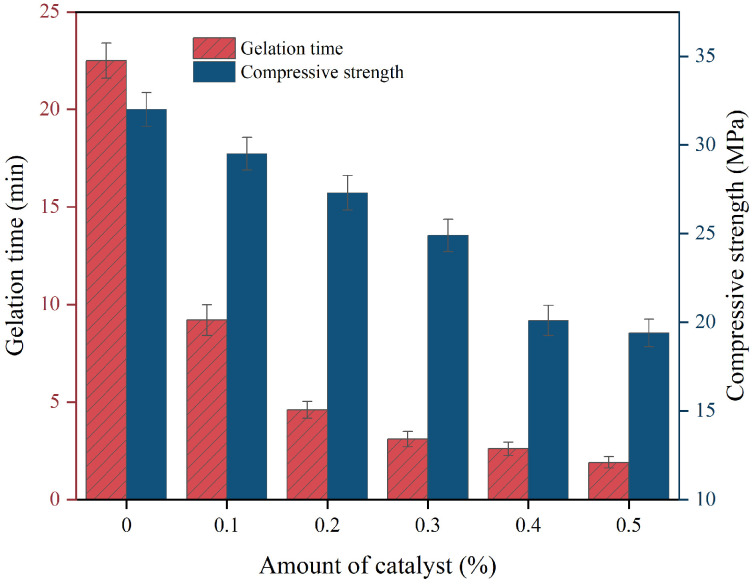
The effect of the amount of catalyst on the gelation time and compressive strength of the composite.

In grouting projects, especially in water plugging grouting in underground mines, slurries with low setting rates are easily washed away by water flow before forming a gel, so the rapid setting performance of slurries has an important influence on the grouting effect. Figure 11 demonstrates the effect of the dosage of organotin catalyst on the setting time and compressive strength of polymer slurry. From Figure 11, it can be seen that the use of a catalyst significantly shortens the gel time of polymer slurry, and the gel time of the material is shortened to 4.6 min at 0.2% catalyst dosage, and when the catalyst dosage is more than 0.2%, the actual catalytic effect is not obvious, and the gel rate of the material grows slowly.

The material’s compressive strength was reduced by the catalyst addition, with a decrease in strength as the catalyst dosage increased. At a 0.5% catalyst dosage, the compressive strength dropped to 19.4 MPa, 39% lower than the sample without a catalyst.

The organotin catalyst, as a Lewis acid, enhances the activity of the isocyanate group by forming a complex with the nitrogen atom on the isocyanate group in the polymer, making it easier to react with the hydroxyl group in the polyol. This accelerating effect directly leads to a significant reduction in gel time. In addition, organotin catalyzes the rapid increase in polymerization and cross-linking within the polyurethane structure, leading to the creation of a denser network structure. The quick development of this network is crucial for gelation, leading to a faster transition from liquid to solid in the system.

The catalyst may negatively affect the compressive strength of the material by speeding up the foaming reaction of the polymer, resulting in an undesirable pore structure with uneven pore sizes and irregular spaces, ultimately impacting the material’s mechanical properties. In addition, the rapid chemical reaction of the polymer can lead to uneven distribution of cross-linking points, which in turn causes stress concentration and makes the material more prone to localized damage when under pressure.

#### 3.8.2. Effect of Coupling Agent on Composite Materials

**Figure 12:**
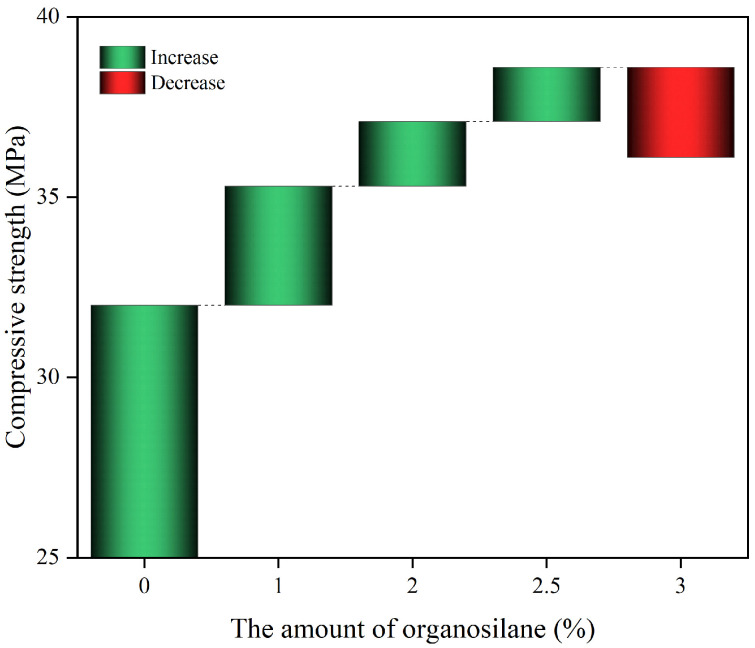
The effect of the amount of organosilane on the compressive strength of the composite.

The impact of adding organosilane on the compressive strength of the polymer composites is shown in Figure 12.

The polymer’s strength rises as more organosilanes are added, peaking at 2.5% with a maximum compressive strength of 38.6 MPa, a 20% increase. However, at 3% organosilanes, the strength growth diminishes noticeably. The reason why organosilanes can positively affect the compressive strength of polymer materials is that the end molecular chain of organosilane molecules and the hydroxyl group on the surface of fly ash generate a chemical bond after a series of chemical reactions, such as dehydration and condensation, while the other end is tightly bonded with the urethane matrix, which results in a significant enhancement of the solid interfacial layer of the material. The addition of organosilane increases the crosslinking degree between fly ash and polymer materials, which in turn improves the compressive properties of the material. Excessive use of organosilanes leads to a reduction in material compressive strength, as the surplus organosilanes undergo continuous hydrolysis on the fly ash surface, resulting in the formation of silanol molecules that impede the polymer matrix cross-linking with the organosilanes. In addition, excessive organosilanes are prone to self-polymerization, which hinders the cross-linking combination between the polymer matrix and fly ash. Hence, if the concentration of organosilanes reaches 3%, the compressive strength of the polymer composites will decrease rather than increase.

## 4. Conclusions

In this paper, a high-efficiency and low-cost polymer composite grouting material in a mine grouting project was prepared by using polyurethane grouting material as the main body, fly ash as the inorganic filler, and auxiliary reagents such as catalyst and coupling agent. Through the research on mechanical properties, thermal stability, hydrophobicity, and adhesion of the composite grouting material, the following conclusions were obtained:

- The addition of fly ash can lower the polymer materials’ reaction temperature, and enhance their thermal stability, hydrophobicity, and mechanical characteristics. However, the bond strength of the composites was reduced due to the fly ash limiting the formation of bond strength of the polymers.
- As a coupling agent, one end of the molecular chain of organosilane and the hydroxyl group on the surface of fly ash generated a chemical bond through dehydration condensation, while the other end was tightly bonded with the polyurethane matrix, resulting in a significant enhancement of the solid interfacial layer of the material. Therefore, the addition of organosilanes plays a positive role in the mechanical properties of polymer composites.
- The use of dibutyltin dilaurate as a catalyst can increase the activity of isocyanate groups so that it is easier to react with the hydroxyl groups in the polyol, which can effectively reduce the gel time of the polymer grouting material.
- The dosage of the coupling agent and catalyst is not the more, the better. Appropriate auxiliary reagents should be configured according to the needs of the project.
- These studies help to ensure the efficiency and economy of mine grouting projects and provide theoretical support and guidance for the application of polymer composites in mine engineering.

## Figures and Tables

**Table 1:** The main composition and proportion of fly ash.

Serial Number	Component	Proportion (%)
1	SiO_2_	48.85
2	Al_2_O_3_	35.72
3	Fe_2_O_3_	4.33
4	CaO	4.28
5	TiO_2_	1.37
6	Others	5.45

**Table 2:** Ingredients and dosage of composite materials.

Raw Material	Type	Proportion (%)
Isocyanate	MDI	35
Polyether Polyol	GE-220	35
Inorganic Filler	UFA	10–30
Coupling Agent	KH-550	1–3
Catalyst	DBTDL	0.1–0.5

## Data Availability

Data are contained within the article.
